# Supplementary material for: Multivariable Behavioral Change Modeling of Epidemics in the Presence of Undetected Infections
Source: Stat Med. 2026 Aug 16;45(18-19):e70709. doi: 10.1002/sim.70709 (PMC13478002; doi:10.1002/sim.70709)
Supplement: Supplementary file 1 [file SIM-45-0-s001.pdf]

Supplementary Material for "Multivariable Behavioral Change Modeling of Epidemics in the Presence of Undetected Infections".

1 SIHRD Model Diagram

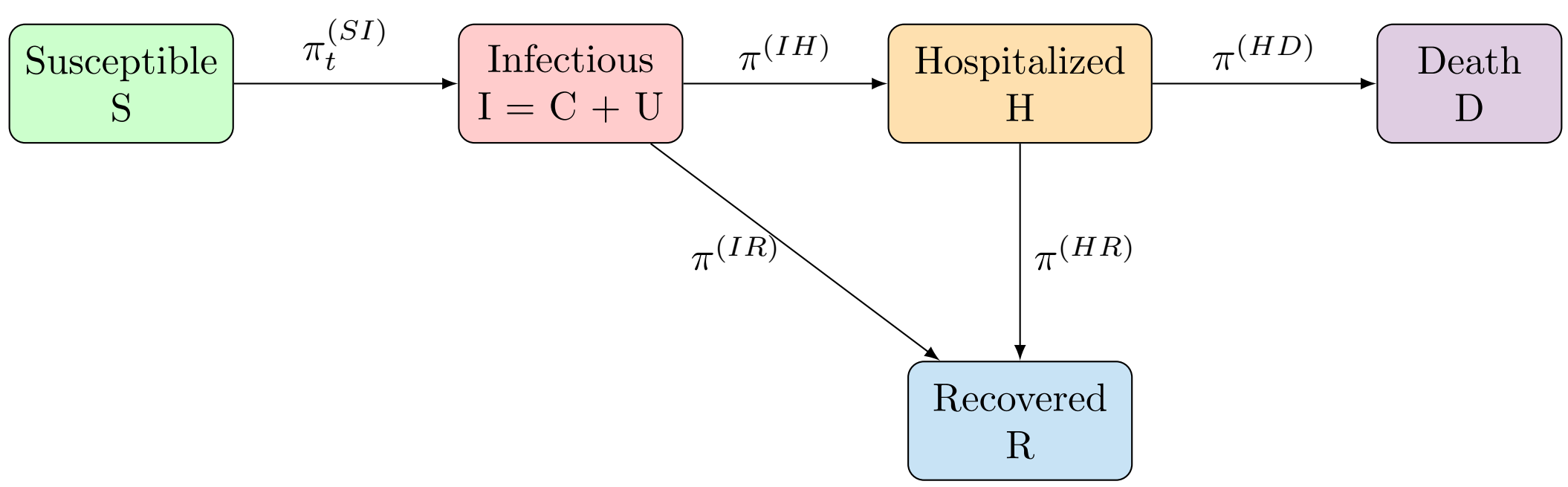

Supplementary Figure 1: Diagram of the full SIHRD model with undetected infections. The simpler SIR model excludes the Hospitalized and Death compartments, and the associated transition probabilities. Models using only detected cases would assume $U = 0$.

1.1 Reproductive Number Calculation

Often, the primary inferential interest in compartmental epidemic models is the reproductive number as it quantifies the magnitude of disease spread in a population. We consider the time-varying "effective" reproductive number, $\mathcal{R}_0(t)$, defined as the expected number of secondary infections caused by a single infected individual in the current population at time t, as our interest is in incorporating behavioral change leading to changing transmission over time. As shown in Ward et al. (2022), this can be generally derived as an expectation from the chain binomial SIR model presented in Section **??** as $\mathcal{R}_0(t) = \sum_{j=t}^{\infty} S_j \pi_{0j}^{(SI)} \left(1 - \pi^{(IR)}\right)^{j-t}$, where for a single individual infected at time t, $\pi_{0j}^{(SI)}$ denotes the probability of transmission from that individual at time j. Intuitively, the formulation is derived from assuming that on any given day, the expected number of secondary infections is $S_j \times \pi_{0j}^{(SI)}$, and then over the course of the infection this quantity is weighted by the probability that the individual is still infectious (i.e., has not become removed). For the IDD transmissibility specification we are using to implement the SIR model, the duration of the infection is fixed so this simplifies to $\mathcal{R}_0(t) = \sum_{j=t}^{t+T_I} S_j \left[1 - \exp\left\{ - \beta(1-a_j)\frac{f(j-t)}{N}\right\}\right]$. This formulation can be directly extended to the SIHRD compartmental structure, however, we must be sure to account for the multinomial transition out of the Infectious compartment as according to the model individuals can stop transmitting the disease when they recover *or* when they become hospitalized. Thus, the effective reproductive number is calculated as $\mathcal{R}_0(t) = \sum_{j=t}^{\infty} S_j \left[1 - \exp\left\{ - \beta(1-a_j)\frac{1}{N}\right\}\right] \left(1 - \pi^{(IH)} - \pi^{(IR)}\right)^{j-t}$.

2 IDD Transmissibility Curves

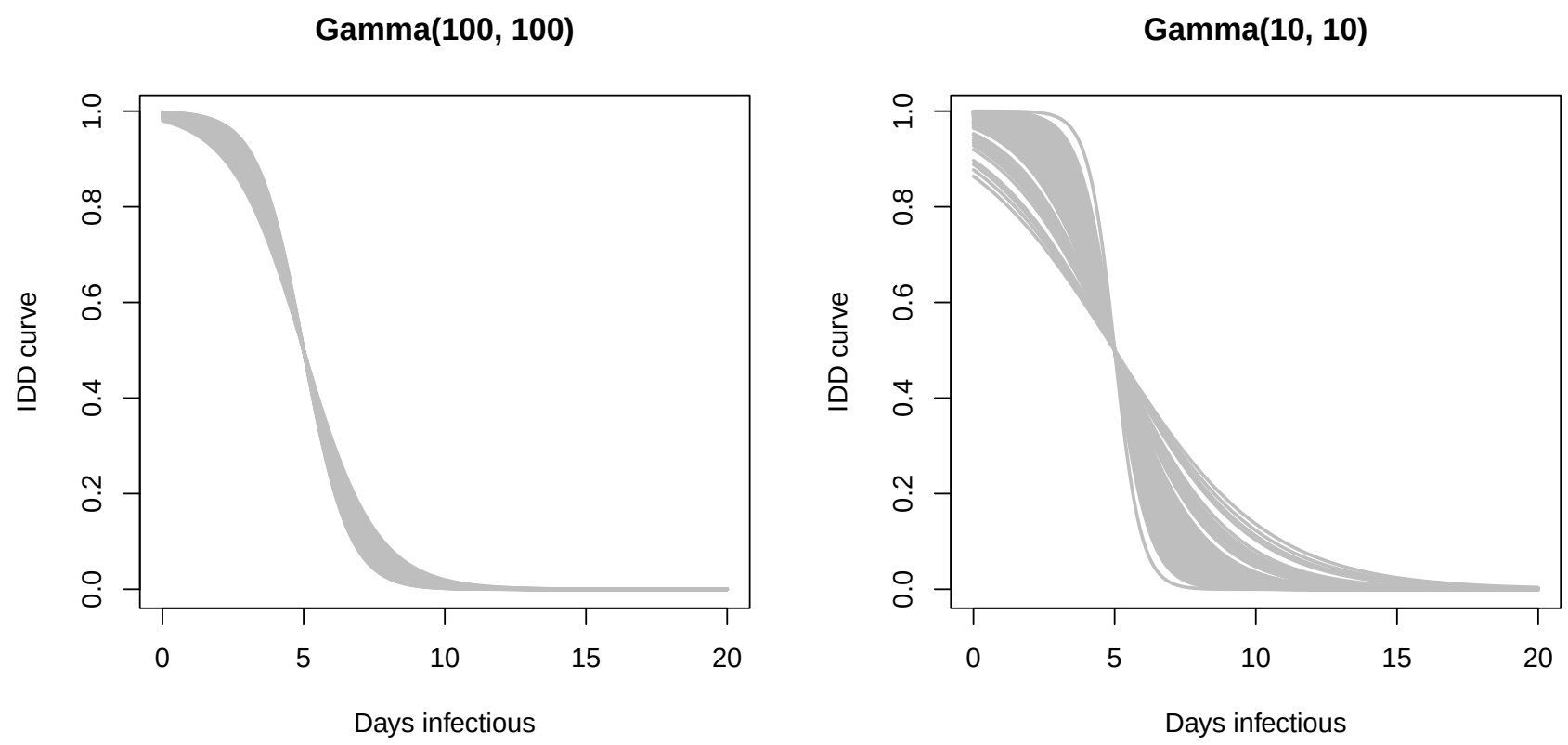

Supplementary Figure 2: Logistic decay IDD transmissibility curves with $w_0 = 5$ and 100 draws from priors for ν from either a Gamma(100,100) prior or a Gamma(10, 10) prior. The Gamma(100,100) was used in our implementation to enforce a sharp decrease in transmission around the inflection point.

3 Simulation Study

3.1 Simulation Set-up

Description	Parameter	High case importance	Equal importance	High death importance
rate for I to R	γ_1	0.2	0.2	0.2
rate for H to R	γ_2	0.2	0.2	0.2
rate for I to H	λ	0.03	0.03	0.03
rate for H to D	ϕ	0.1	0.1	0.1
case detection probability	π^{detect}	0.25	0.25	0.25
baseline transmission	β	1.5	1.5	1.5
overall alarm parameter	k	0.00003	0.00003	0.00003
relative importance of cases	α	0.85	0.50	0.15

Supplementary Table 1: Parameters used in simulation data generation

Description	Parameter	SIHRD	SIR
rate parameter for I to R	γ_1	$\Gamma(2000, 10000)$	-
rate parameter for H to R	γ_2	$\Gamma(2000, 10000)$	-
rate parameter for I to H	λ	$\Gamma(300, 10000)$	-
rate parameter for H to D	ϕ	$\Gamma(100, 1000)$	-
cases detection probability	π^{detect}	$\text{Beta}(2500, 7500)$	$\text{Beta}(2500, 7500)$
baseline transmission	β	$\Gamma(0.1, 0.1)$	$\Gamma(0.1, 0.1)$
overall alarm parameter	k	$\Gamma(0.1, 0.1)$	$\Gamma(0.1, 0.1)$
relative importance of cases in alarm	α	$U(0, 1)$	$U(0, 1)$
midpoint IDD curve	w_0	-	$N(5, 0.1^2)$
IDD decay rate	ν	-	$\Gamma(1000, 1000)$

Supplementary Table 2: Prior distributions used for all parameters in simulation study. Only models with cases and deaths in the alarm estimate α, and models with no alarm also do not include k. Models which ignore undetected infections do not include π^{detect}.

3.2 Estimation of $R_0(t)$ across all time points

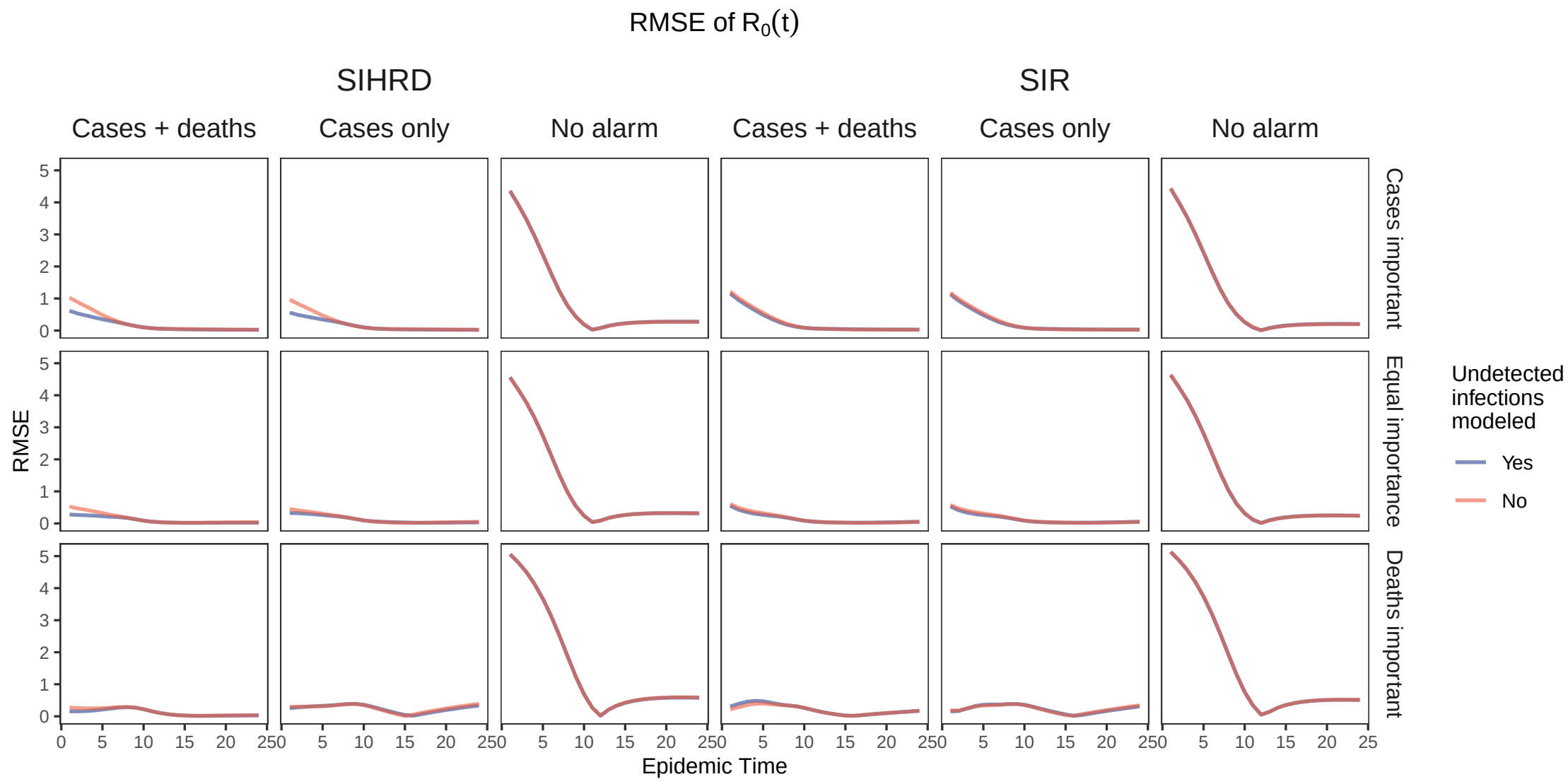

Supplementary Figure 3: Root mean squared error (RMSE) of the baseline effective reproductive number across epidemic time.

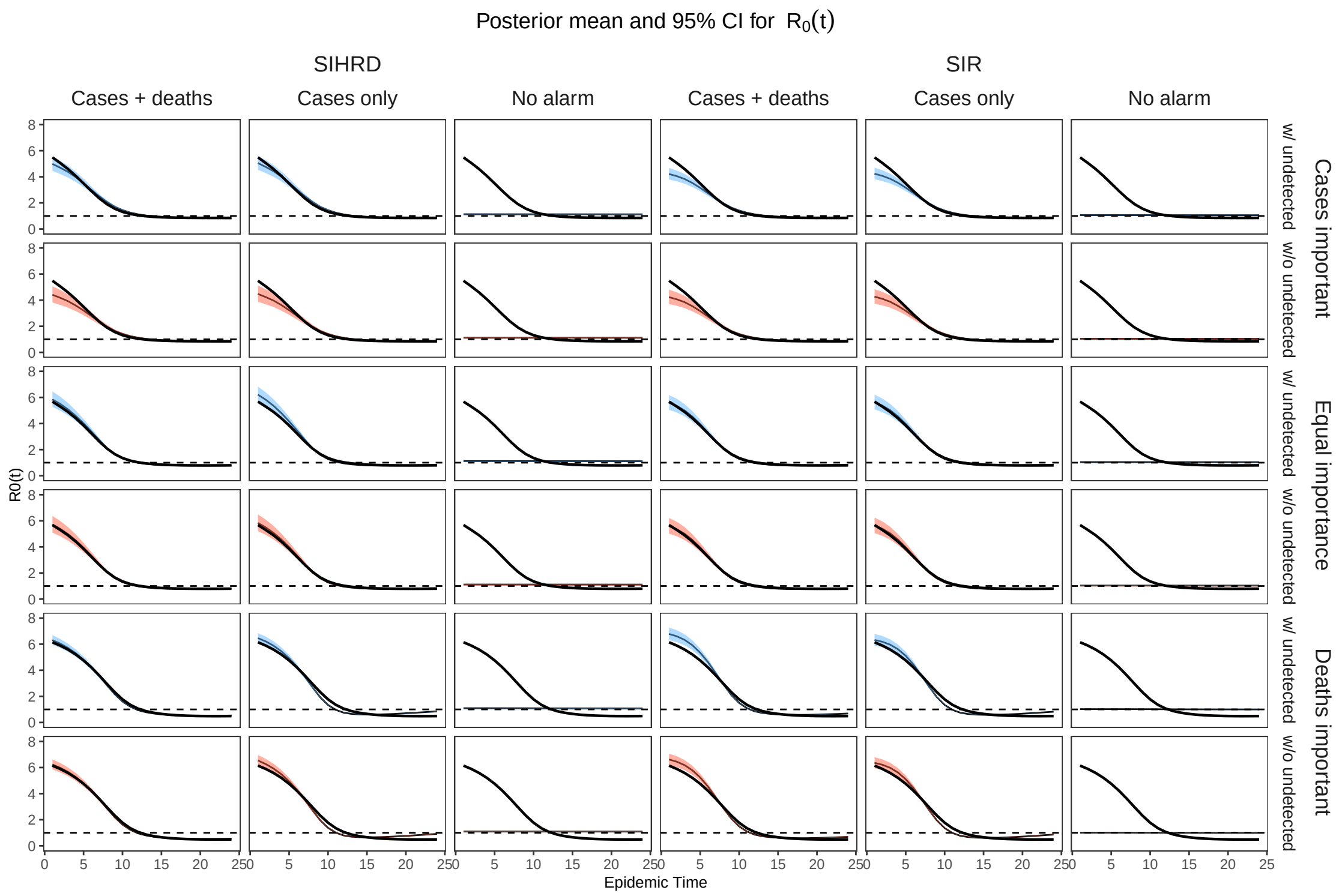

Supplementary Figure 4: Posterior mean and 95% credible intervals for $\mathcal{R}_0(t)$ for one randomly selected simulated epidemic in each data generating scenario

3.3 Posterior distributions of all parameters

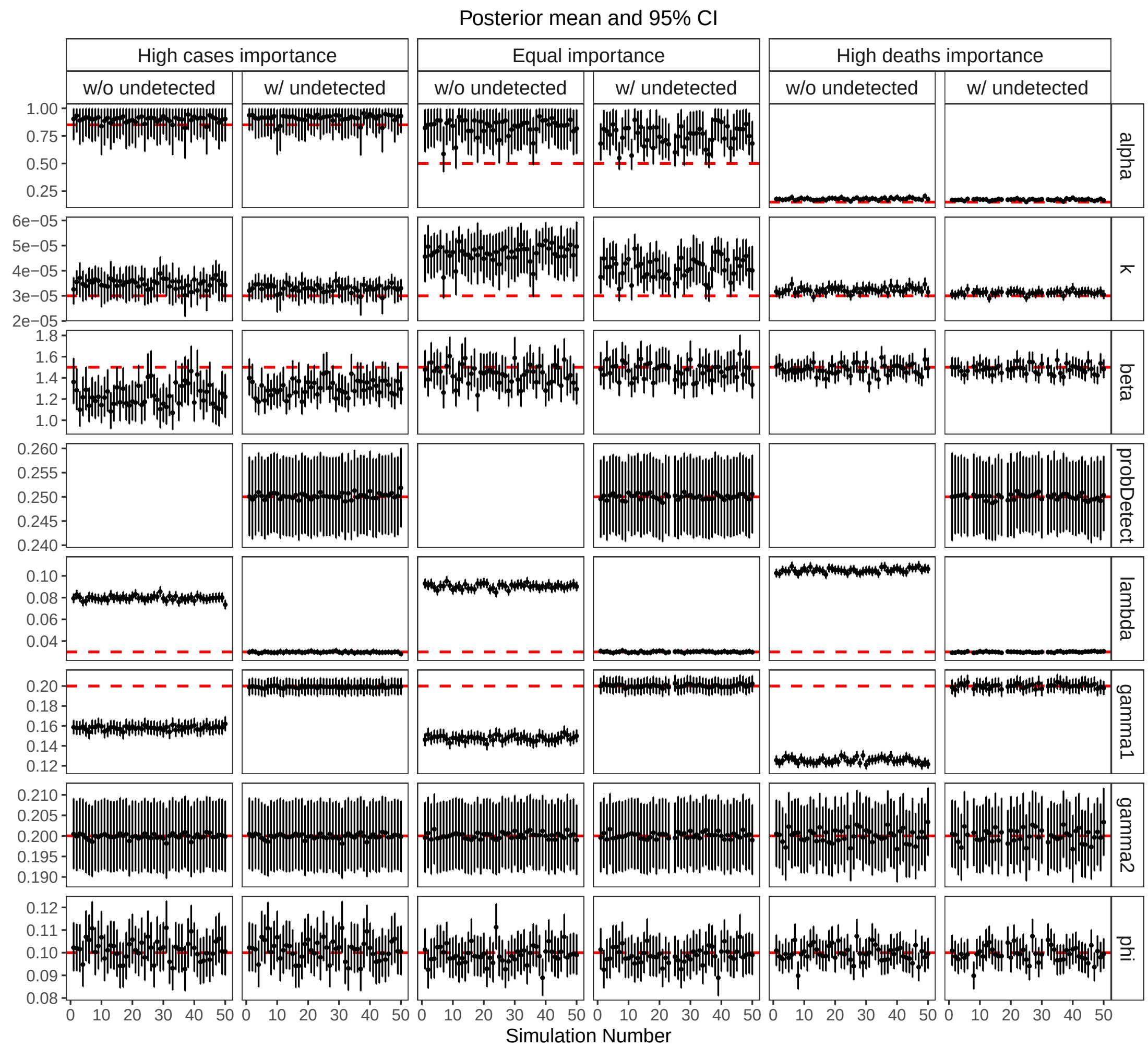

Supplementary Figure 5: Posterior means and 95% credible intervals for parameters in the SIHRD models with cases and deaths in the alarm function

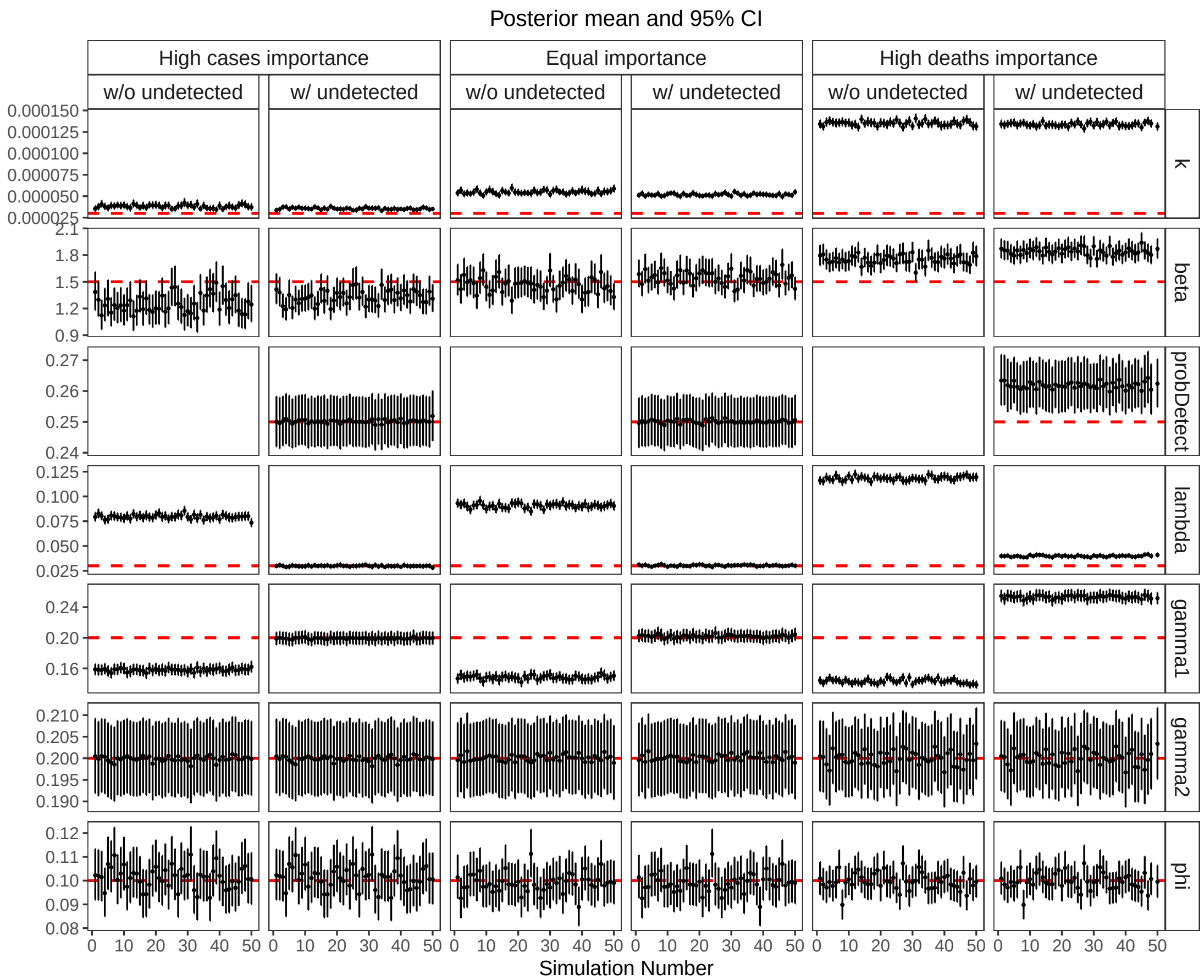

Supplementary Figure 6: Posterior means and 95% credible intervals for parameters in the SIHRD models with cases only in the alarm function

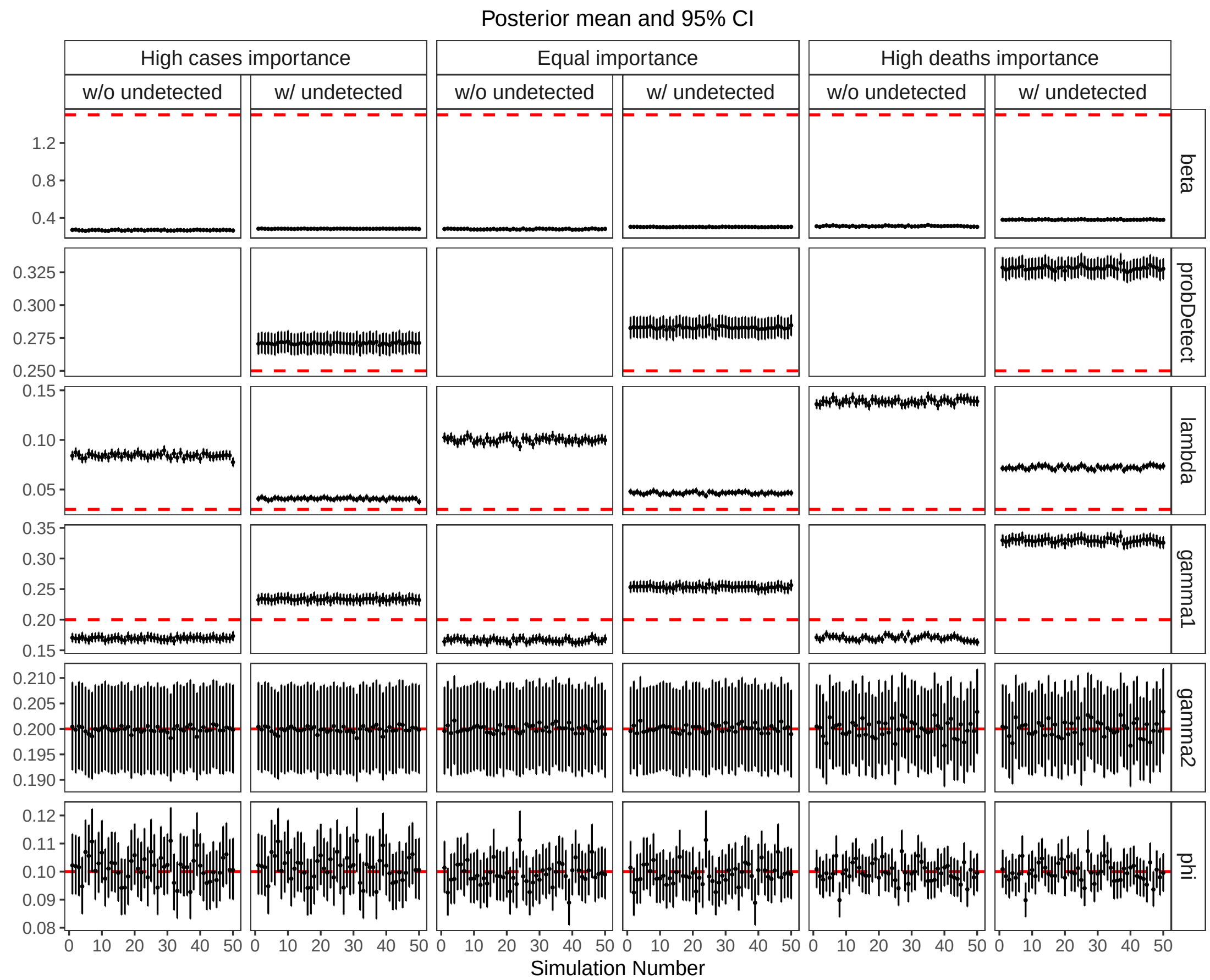

Supplementary Figure 7: Posterior means and 95% credible intervals for parameters in the SIHRD models with no alarm function

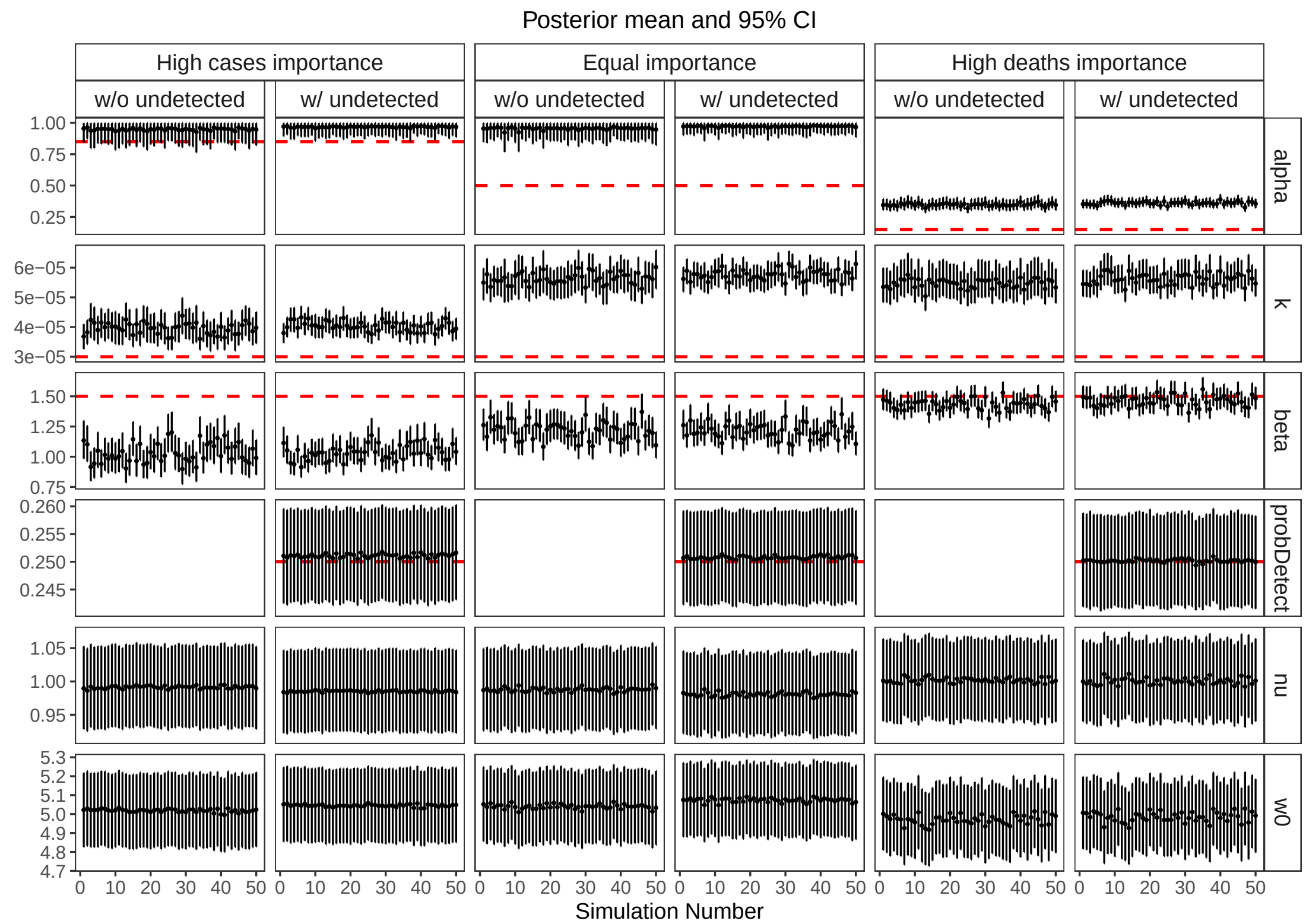

Supplementary Figure 8: Posterior means and 95% credible intervals for parameters in the SIR models with cases and deaths in the alarm function

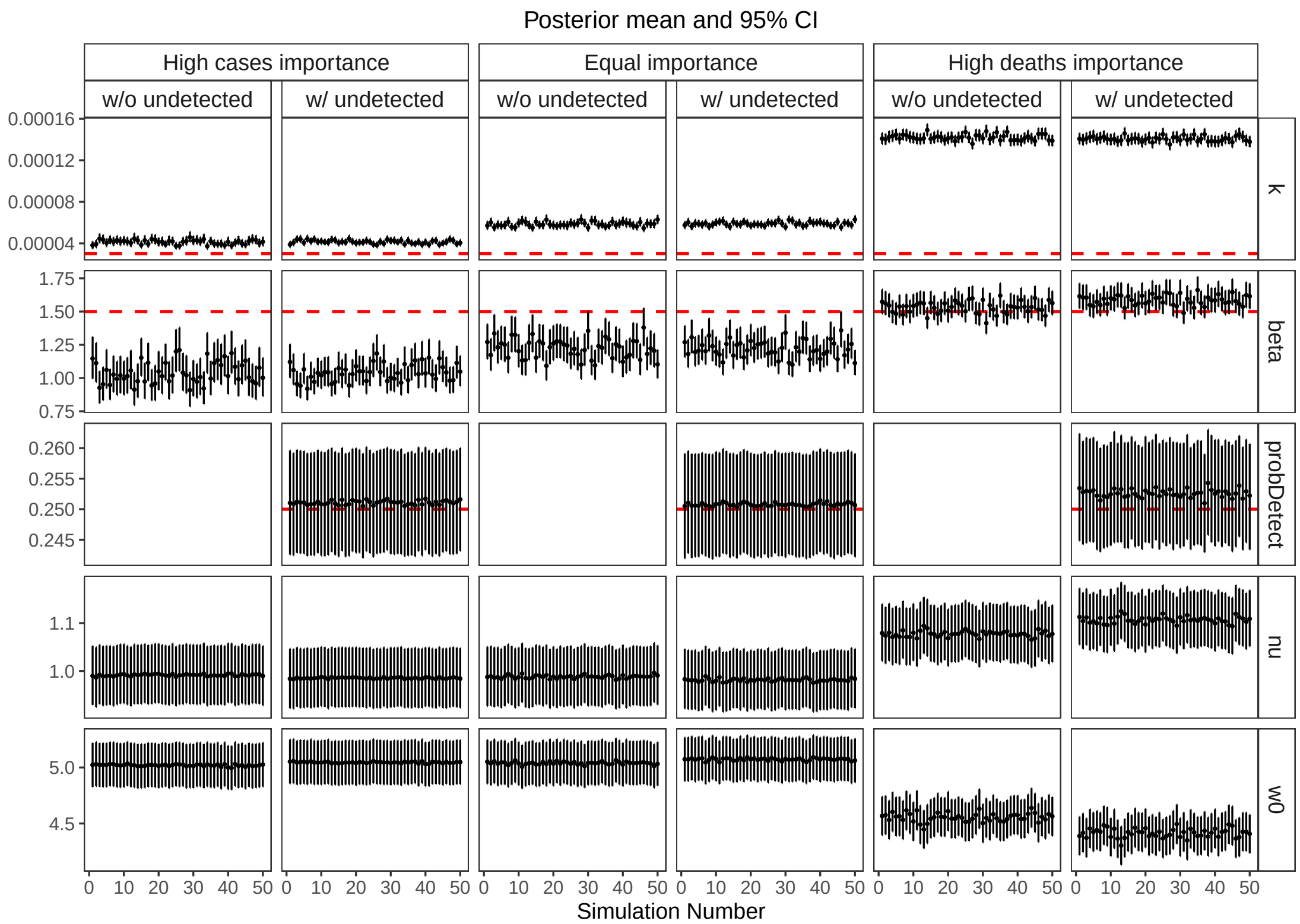

Supplementary Figure 9: Posterior means and 95% credible intervals for parameters in the SIR models with cases only in the alarm function

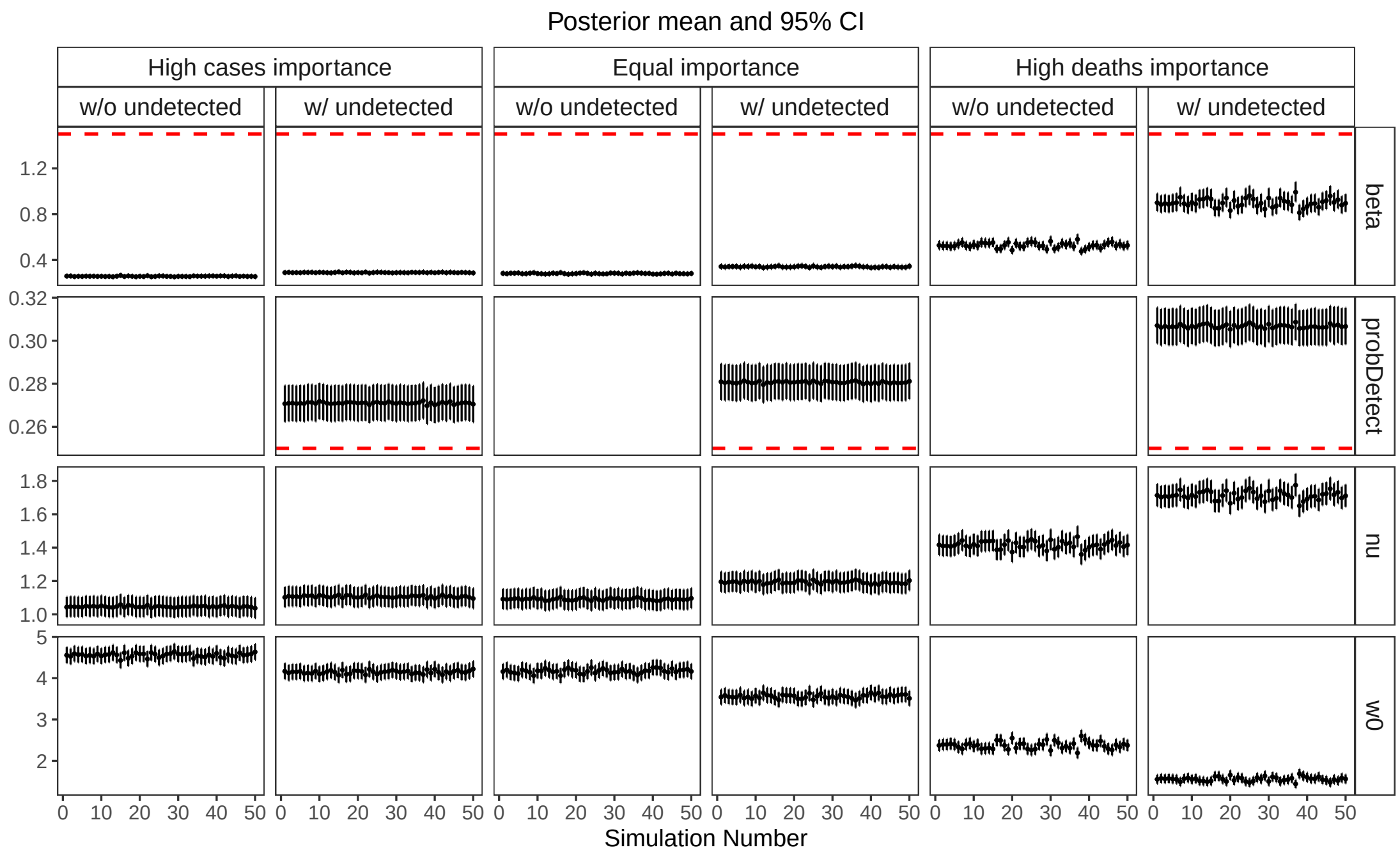

Supplementary Figure 10: Posterior means and 95% credible intervals for parameters in the SIR models with no alarm function

4 Supplemental Simulations Assuming Hospitalizations Only Arise From Detected Infections

4.1 Set-up

Our model implementation in the main text assumed that hospitalizations could arise from both detected and undetected infections, i.e., that

$$\{H_t^*, R_t^{I^*}, I_{t+1}\} \sim Multinom\left(I_t, \left\{\pi^{(IH)}, \pi^{(IR)}, 1-\pi^{(IH)}-\pi^{(IR)}\right\}\right),$$

with $\pi^{(IH)} = 1-\exp(-\lambda)$ and $\pi^{(IR)} = 1-\exp(-\gamma_1)$. This increased realism requires strong priors on γ_1 and λ to induce identifiability, as otherwise similar observed hospitalization data could arise from a low detection probability and high hospitalization rate (λ) or a high detection probability and lower hospitalization rate. Alternatively, one could consider a model where hospitalizations only arise from the detected case counts, i.e., the full modified model specification is:

$$\begin{aligned}
I_t^* &\sim Binom\left(S_t, \pi_t^{(SI)}\right)\\
C_t^* &\sim Binom\left(I_t^*, \pi^{detect}\right)\\
H_t^* &\sim Binom\left(C_t, \pi^{(IH)}\right)\\
R_t^{I^*} &\sim Binom\left(I_t - H_t^*, \pi^{(IR)}\right)\\
\{D_t^*, R_t^{H^*}, H_{t+1}\} &\sim Multinom\left(H_t, \left\{\pi^{(HD)}, \pi^{(HR)}, 1-\pi^{(HD)}-\pi^{(HR)}\right\}\right),
\end{aligned}$$

with slightly modified difference equations to include updates of the total number of detected cases at each time point,

$$\begin{aligned}
S_{t+1} &= S_t - I_t^*\\
C_{t+1} &= C_t - C_t^* - H_t^*\\
I_{t+1} &= I_t + I_t^* - H_t^* - R_t^{I^*}\\
H_{t+1} &= H_t + H_t^* - R_t^{H^*} - D_t^*\\
R_{t+1} &= R_t + R_t^{I^*} + R_t^{H^*}\\
D_{t+1} &= D_t + D_t^*.
\end{aligned}$$

This model formulation also changes the specification of the reproductive number as the calculation of $\mathcal{R}_0(t)$ must account for the transition out of the Infectious compartment. For the model in the main text with a multinomial transition out of the infectious compartment calculating the probability of remaining infectious is straightforward as $1-\pi^{(IH)}-\pi^{(IR)}$. For the modified model, individuals remain infectious if they are either not detected *and* then subsequently not hospitalized, or if they are not removed. However, removal is conditional on the set of infectious individual that are no longer hospitalized. Therefore, the probability of hospitalization is $P(H) = P(H|C)P(C) = \pi^{(IH)}\pi^{detect}$. Removal can occur for those that are detected or not detected, but for those detected only those that are not hospitalized are eligible for removal. Therefore, the probability of removal is

$$\begin{aligned}
P(R) &= P(R|C\cap H^C)P(H^C|C)P(C) + P(R|C^C)P(C^C)\\
&= \pi^{(IR)}(1-\pi^{(IH)})\pi^{detect} + \pi^{(IR)}(1-\pi^{detect})\\
&= \pi^{(IR)}[(1-\pi^{(IH)})\pi^{detect} + (1-\pi^{detect})]\\
&= \pi^{(IR)}[\pi^{detect} - \pi^{(IH)}\pi^{detect} + 1 - \pi^{detect}]\\
&= \pi^{(IR)}[1-\pi^{(IH)}\pi^{detect}].
\end{aligned}$$

Combining, the probability of an individual staying infectious in this model is

$$1 - \pi^{(IH)}\pi^{detect} - \pi^{(IR)}[1 - \pi^{(IH)}\pi^{detect}]$$

which simplifies to

$$\left(1 - \pi^{(IR)}\right)\left(1 - \pi^{(IH)}\pi^{detect}\right).$$

Thus, the effective reproductive number is calculated as

$$\mathcal{R}_0(t) = \sum_{j=t}^{\infty} S_j\Big[1 - \exp\Big\{-\beta(1 - a_j)\frac{1}{N}\Big\}\Big]\Big[\left(1 - \pi^{(IR)}\right)\left(1 - \pi^{(IH)}\pi^{detect}\right)\Big]^{j-t}.$$

To investigate whether this modeling approach relaxed the need for strong priors on λ and whether it would change our primary conclusions, we performed a similar simulation study as in the main text. The data was generated from this modified model and we consider only the case where undetected infections are incorporated in the SIHRD model structure. All parameters were set to the same values as described in Supplementary Table 1 and all priors used were the same as described in Supplementary Table 2 except the prior for λ, which was relaxed from $\lambda \sim \Gamma(300, 10000)$ to $\lambda \sim \Gamma(3, 100)$. We also tried relaxing the prior on γ_1, however, we found that particularly in the high deaths scenario there was high correlation between γ_1 and π^{detect}, which often inflated the Gelman-Rubin diagnostic to be larger than 1.1.

4.2 Results

Overall, the data generated from this modified model looks very similar to the data generated from our primary SIHRD model (Supplementary Figure 11). The modified model was able to achieve satisfactory convergence with the relaxed prior on λ as expected. In the high deaths scenario, across all the SIHRD models fitted, the Gelman-Rubin diagnostic was often above 1.1 (typically between 1.1 and 1.5) for π^{detect}. Visual inspection of the trace plots showed that autocorrelation was leading to these larger values but all parameters exhibited stationarity so these simulations are included in our results. In replicating the results of the simulation presented in the main text (Supplementary Figures 12-14), we see minimal differences between the modified SIHRD model and our implemented version in the main text. This suggests that the modified model with hospitalizations only arising from the observed cases counts is a reasonable approach that improves the identifiability of the parameter λ and does not seem to results in any severe consequences in terms of estimation of the reproductive number or alarm function parameters. However, in practice it is difficult to assess whether this assumption truly hold or not as real-world data do not provide a link between individuals in the case counts and hospitalization.

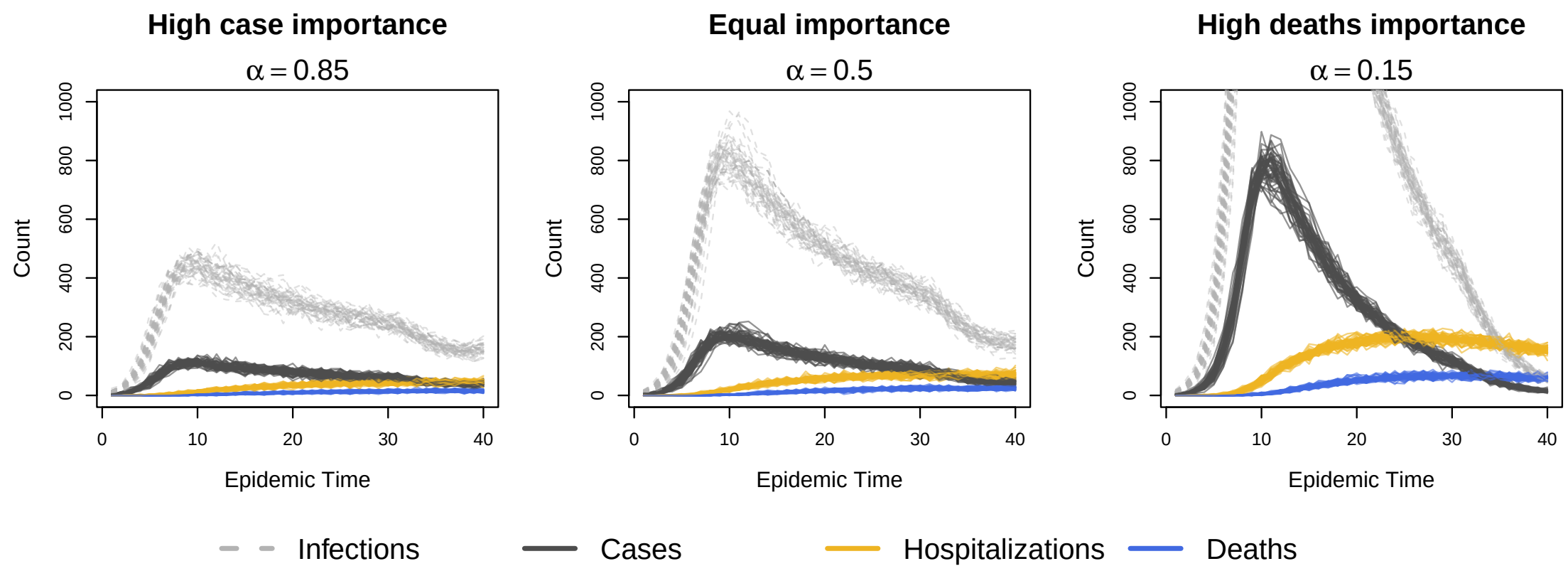

Supplementary Figure 11: Simulated epidemics from the modified SIHRD model with three scenarios capturing various importance of cases and deaths informing behavioral change. Plotted over time are the total (unobserved) infections (dashed gray), observed cases (solid black), hospitalizations (solid gold), and deaths (solid blue). In the high deaths importance scenario, infections peak at counts around 3,000 but this was excluded from the figure to highlight more subtle differences between high case importance and equal importance generating scenarios. This figure corresponds with Figure 1 in the main text.

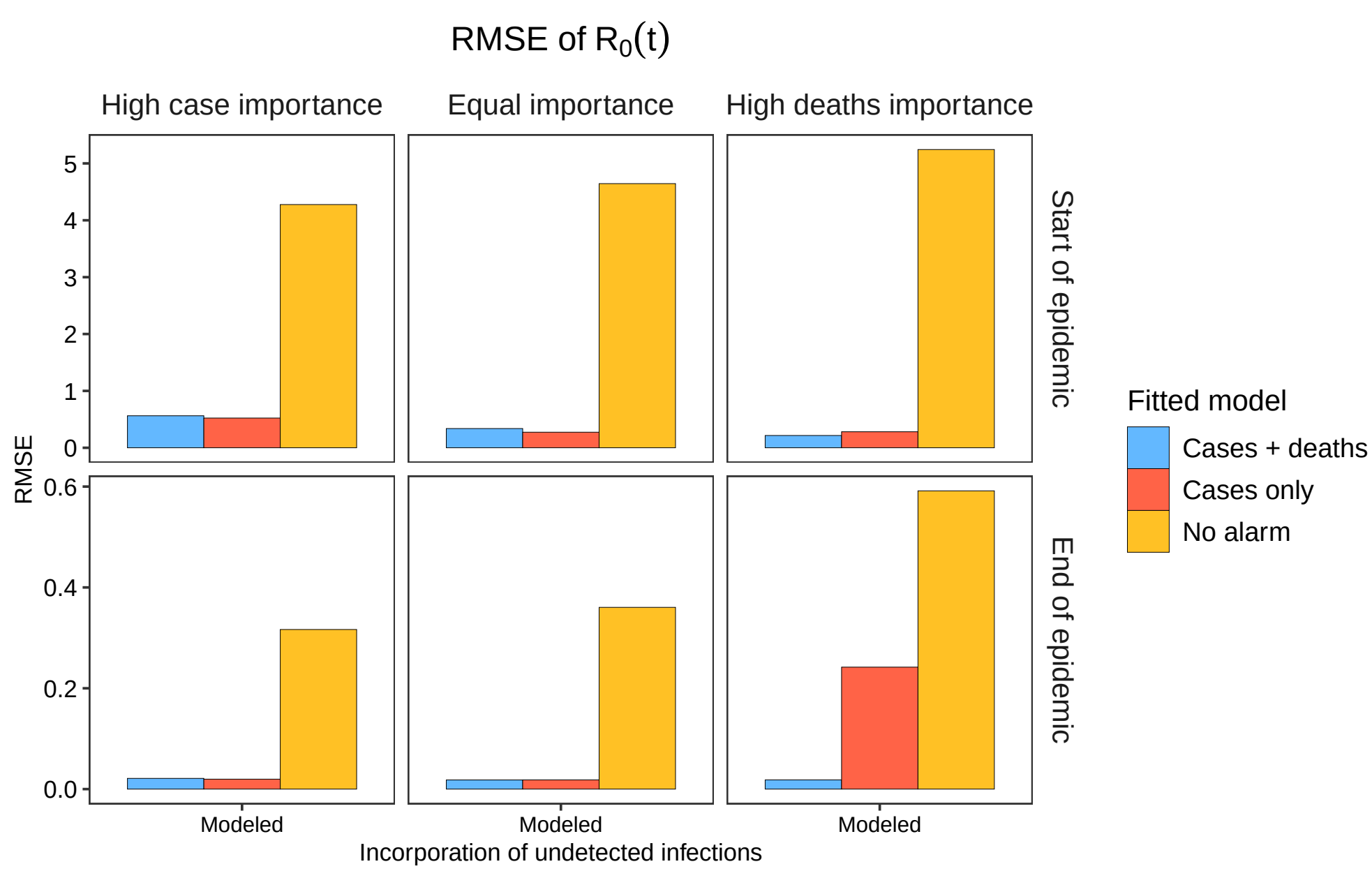

Supplementary Figure 12: Root mean squared error (RMSE) of the effective reproductive number, $\mathcal{R}_0(t)$, at the start and end of the simulated epidemics for the three data generating scenarios for the modified SIHRD model modeling undetected infections. This figure corresponds with Figure 2 in the main text.

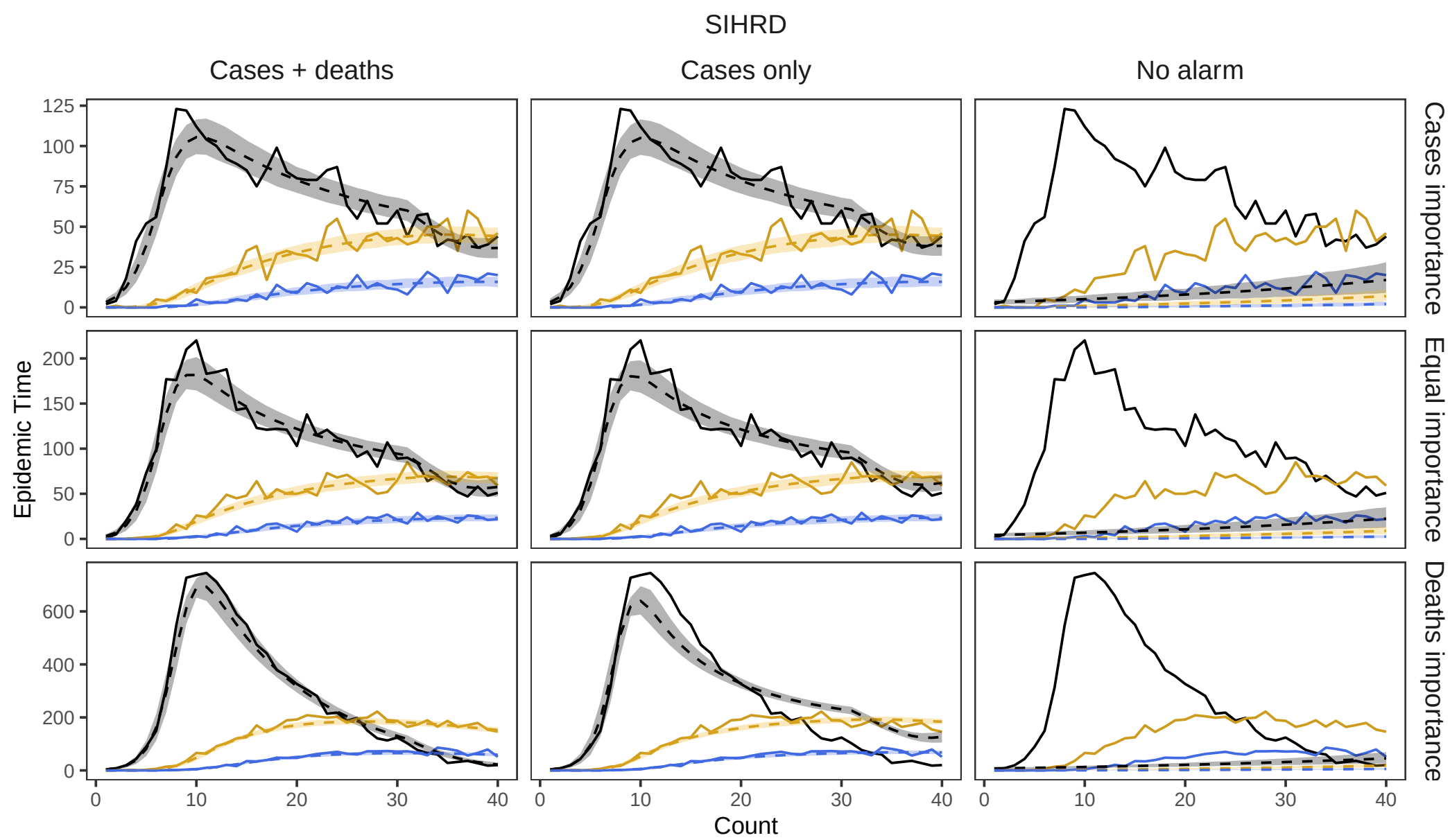

Supplementary Figure 13: Posterior predictive means (dashed lines) and 95% credible intervals (shaded regions) for one randomly selected simulation compared to true cases (solid black), hospitalizations (solid gold), and deaths (solid blue). These results are for the modified SIHRD model modeling undetected infections. This figure corresponds with Figure 4 in the main text.

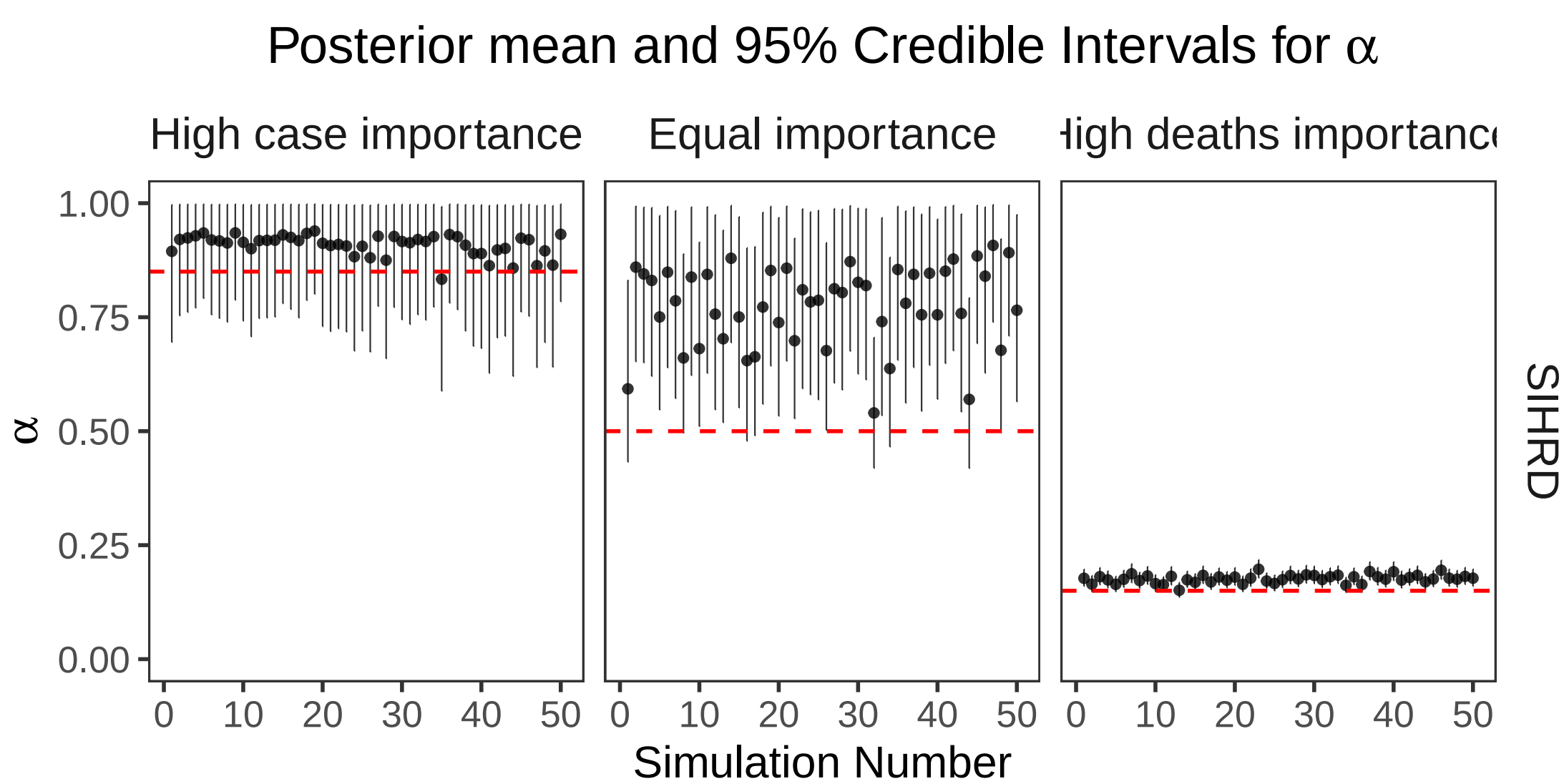

Supplementary Figure 14: Posterior means and 95% credible intervals for α across all simulations for the three data generating scenarios (columns) and the modified SIHRD model with multivariable behavioral change. The true parameters used in simulation are shown by the dashed red lines. These results are for the modified SIHRD model modeling undetected infections. This figure corresponds with Figure 4 in the main text.

5 COVID-19 Data Analysis

5.1 Prior specifications

The prior on the probability of case detection, π^{detect} varied by city and by wave. As described in the main text, the prior means for Miami were 12% in wave 1 and 34% in wave 2 and for Montréal, were 25% in wave 1 and 40% in wave 2. The exact priors used are specified in Table 3. A stronger prior was required during Wave 2 in Miami due to the exceptionally large number of cases reported during that wave.

	Wave 1	Wave 2
Miami	Beta(1200, 8800)	Beta(34000, 66000)
Montréal	Beta(2500, 7500)	Beta(4000, 6000)

Supplementary Table 3: Prior distributions used for the detection probability, π^{detect}, in both Miami and Montréal data analyses.

The priors for the initial values in each compartment, $Y_0 = \{S_0, I_0, H_0, R_0, D_0\}$ or $Y_0 = \{S_0, I_0, R_0\}$, were assumed to be multinomially distributed as either $Y_0 \sim Multinom(N, \{\bar{S}_0, \bar{I}_0, \bar{H}_0, \bar{R}_0, \bar{D}_0\}/N)$ or $Y_0 \sim Multinom(N, \{\bar{S}_0, \bar{I}_0, \bar{R}_0\}/N)$, where $\bar{S}_0$ indicates the estimated number of susceptibles at the start of the epidemic and the values for the other compartments are defined similarly. These estimates are based on the observed data. New cases reported in the previous week divided by the prior mean for case detection determined $\bar{I}_0$ and the new hospitalizations over the previous 15 days determined $\bar{H}_0$. The expected number of deaths at the start of the wave, $\bar{D}_0$, was based on all deaths occurring prior to the first time point. For analysis of the first wave, $\bar{R}_0$ was considered to be 0 corresponding to no previous infections. In the second wave, $\bar{R}_0$ was based on the total number of previous cases occurring more than seven days prior to the start of the wave (i.e., not counted in $\bar{I}_0$) divided by the prior mean for case detection. We did not allow for waning immunity due to the short time period between waves in our analyses, but this could be incorporated by only including infections within some recent time period (e.g., within the past six months) in estimating $\bar{R}_0$, rather than all previous infections.

		SIHRD models					SIR models		
		$\bar{S}_0$	$\bar{I}_0$	$\bar{H}_0$	$\bar{R}_0$	$\bar{D}_0$	$\bar{S}_0$	$\bar{I}_0$	$\bar{R}_0$
Miami	Wave 1	2,701,750	17	0	0	0	2,701,750	17	0
	Wave 2	2,629,688	3,382	448	67,145	1,104	2,629,688	3,382	68,697
Montreal	Wave 1	1,762,935	14	0	0	0	1,762,935	14	0
	Wave 2	1,694,812	425	32	64,443	3237	1,694,812	425	67,712

Supplementary Table 4: Prior distributions used for estimating the initial numbers in each compartment.

Description	Parameter	SIHRD	SIR
rate parameter for I to R	γ_1	$\Gamma(14290, 100000)$	-
rate parameter for H to R	γ_2	$\Gamma(6700, 100000)$	-
rate parameter for I to H	λ	$\Gamma(1000, 100000)$	-
rate parameter for H to D	ϕ	$\Gamma(500, 10000)$	-
baseline transmission	β	$\Gamma(0.1, 0.1)$	$\Gamma(0.1, 0.1)$
overall alarm parameter	k	$\Gamma(0.1, 0.1)$	$\Gamma(0.1, 0.1)$
relative importance of cases in alarm	α	$Unif(0, 1)$	$Unif(0, 1)$
midpoint IDD curve	w_0	-	$N(7, 0.1^2)$
IDD decay rate	ν	-	$\Gamma(1000, 1000)$

Supplementary Table 5: Prior distributions used for remaining parameters in both city's data analyses. Only models with cases and deaths in the alarm estimate α, and models with no alarm also do not include k.

5.2 Sensitivity analysis for weeks of data used

For a few of the SIHRD models fitted to Miami data, the Gelman Rubin (GR) diagnostic value was slightly above 1.1 for the detection probability and/or the rate parameter for the I to H transition (λ). Although visual inspection of the trace plots showed stationarity, there was high autocorrelation leading to the elevated GR diagostic values. To be conservative we have not included the results of those models.

**City**	**Model fitted**	**Weeks used**	**Wave 1**	**Wave 2**
miami	SIHRD	4	0.527 (0.052, 0.977)	-
		6	0.875 (0.619, 0.996)	-
		8	Did not converge	0.000 (0.000, 0.000)
		10	-	0.000 (0.000, 0.000)
		12	-	Did not converge
	SIR	4	0.589 (0.114, 0.981)	-
		6	0.881 (0.636, 0.996)	-
		8	0.949 (0.826, 0.999)	0.456 (0.026, 0.972)
		10	-	0.002 (0.001, 0.003)
		12	-	0.377 (0.069, 0.954)
montreal	SIHRD	6	0.894 (0.664, 0.997)	-
		8	0.941 (0.800, 0.998)	0.001 (0.000, 0.002)
		10	0.965 (0.876, 0.999)	0.455 (0.023, 0.972)
		12	-	0.706 (0.282, 0.988)
	SIR	6	0.899 (0.677, 0.997)	-
		8	0.942 (0.805, 0.998)	0.306 (0.003, 0.958)
		10	0.965 (0.878, 0.999)	0.615 (0.148, 0.983)
		12	-	0.743 (0.344, 0.990)

Supplementary Table 6: Posterior mean and 95% credible intervals for α using various weeks of data for modeling. Bold values are those presented in the main manuscript.

5.3 Posterior predictive distribution of hospitalizations and deaths for SIHRD models

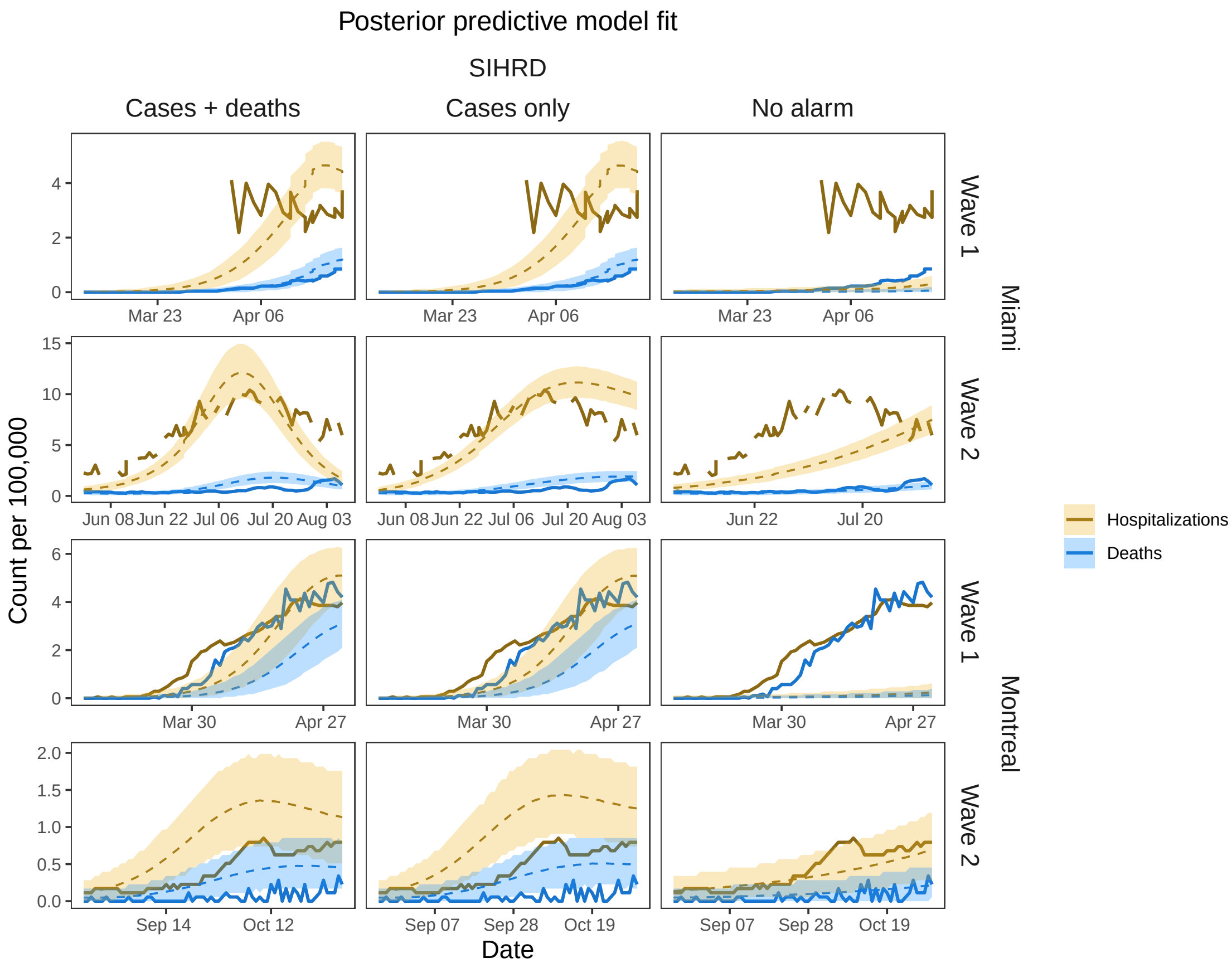

Supplementary Figure 15: Posterior predictive means (dashed lines) and 95% credible intervals (shaded regions) for hospitalizations and deaths compared to observed hospitalizations (solid gold) and deaths (solid blue) for two COVID-19 waves in Miami and Montréal. Hospitalizations and deaths are only shown for the SIHRD model as posterior prediction of deaths is not possible from the SIR model (see Section 2.5 in the main text). Hospitalization data for Wave 1 in Miami was only available from April 2, 2020 and was otherwise imputed.

5.4 Posterior predictive distributions for models without behavioral change

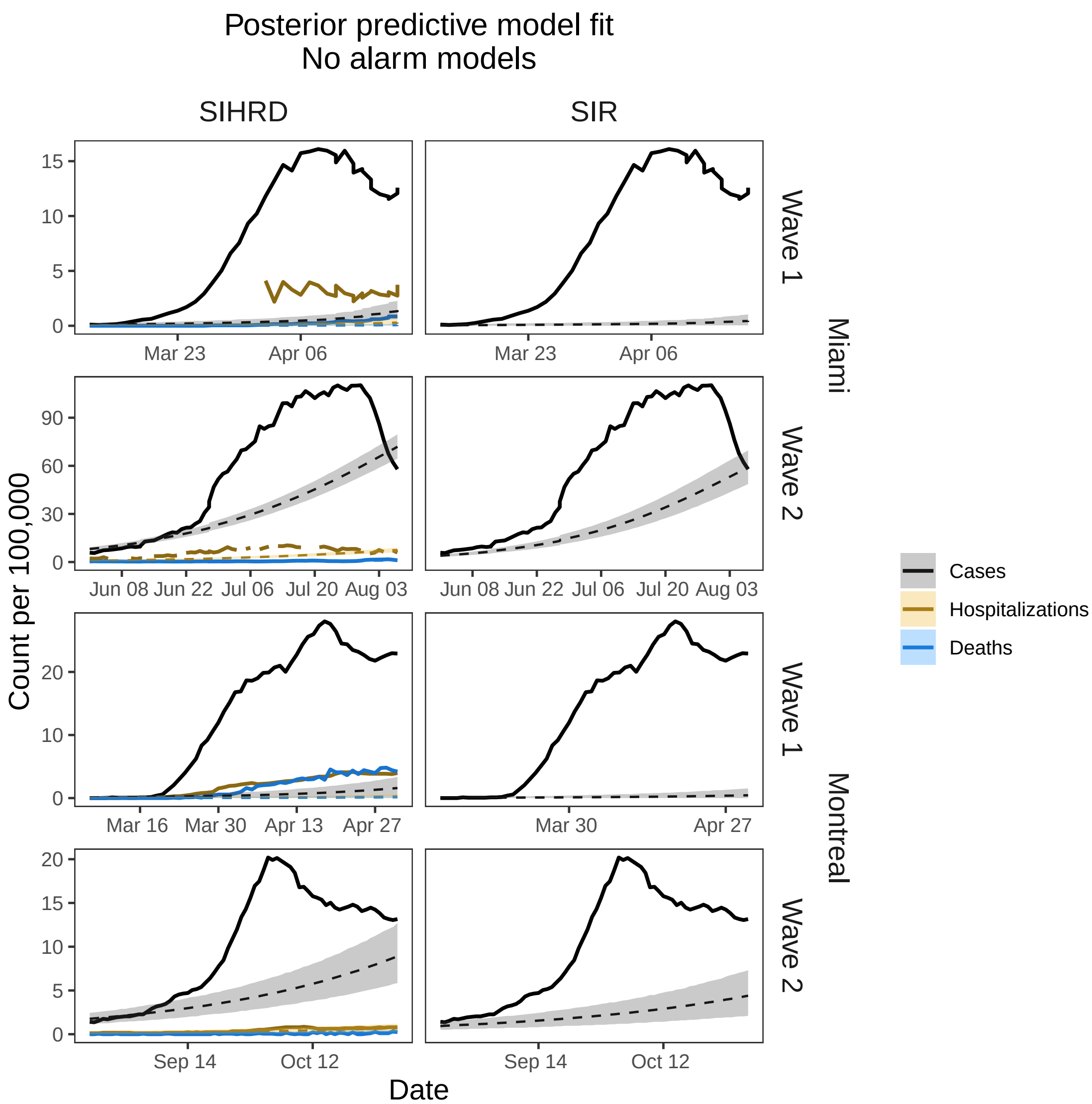

Supplementary Figure 16: Posterior predictive means (dashed lines) and 95% credible intervals (shaded regions) for cases, hospitalizations, and deaths compared to observed cases (solid black), hospitalizations (solid gold), and deaths (solid blue) for two COVID-19 waves in Miami and Montréal for models without behavioral change (no alarm). Hospitalizations and deaths are only shown for the SIHRD model as posterior prediction of deaths is not possible from the SIR model (see Section 2.5 in the main text). Hospitalization data for Wave 1 in Miami was only available from April 2, 2020 and was otherwise imputed.

6 Alternate Alarm Function Specifications

Here we have considered a one-parameter growth function to describe the population alarm, but other functional forms could also be used. Functions used to describe the alarm must satisfy the following properties: for all x, $f(x) \in [0,1]$, $f(0) = 0$, and for $x \leq y$, $f(x) \leq f(y)$. In words, the alarm can only take values between 0 and 1, must be 0 when there is no disease in the population, and is monotonically increasing as disease incidence increases. In addition to the one-parameter growth function we considered here, Ward et al. (2023) considered two other parametric functions which satisfy this criteria. A two-parameter constant change point model $f(x) = \delta \mathbb{1}(x > H)$ with $H \in (0, \max(x))$ $\delta \in [0,1]$, and a three-parameter modified Hill equation $f(x) = \frac{\delta}{1+(x_0/x)^\nu}$ with $\delta \in [0,1]$, $x_0 \in (0, \max(x))$, and $\nu > 0$ which can have a logistic looking shape to represent a smoothed version of the change point function. In considering multivariable extensions of these parametric functions, one must carefully think about identifiability of the overall alarm at any given time point. Both the change point and Hill functions rely on parameters estimating an asymptote (δ) and a mid-point (H or x_0) indicating the value of the observed data where the alarm is increasing. While this allows these functions to be more flexible, these parameterizations lead to identifiability issues when considering multiple data sources as the same epidemic could occur under various specifications of when the alarm components associated with cases or deaths "went off" (reaches the asymptote) and what level the alarm reaches based on each source (value of the asymptote). For example, an alarm fully attributed to cases may go off once smoothed incidence reaches 100 cases per day, but could also be fully attributed to deaths which reached three deaths at the same time. Or the alarm could be equally attributed to cases and death reaching these thresholds. This is problematic as all three scenarios could lead to the same alarm value as a function of epidemic time.

Alternatively, one may consider non-parametric approaches, such as a multivariate Gaussian process or combination of spline functions. However, care must be taken to ensure the final alarm is constrained between 0 and 1, which can be complicated when using multiple inputs. Additionally, one advantage of the current approach is the interpretability of the α parameter as the relative importance of one data source informing population alarm and its not obvious how to translate this interpretation to a non-parametric approach. Another possible approach would be the use of Bayesian model averaging, where each model contains a more complex alarm function based on a single data source and the posterior probabilities of each model would provide the relative importance of the data sources. We plan to pursue both the non-parametric and model averaging approaches as future work to extend this methodology.
